# Hemodynamic Effect of IgM-Enriched Immunoglobulin in the Early Stage of *E. coli*-Induced Experimental Sepsis

**DOI:** 10.3390/jcm15041522

**Published:** 2026-02-14

**Authors:** Balázs Ujhelyi, Ádám Attila Mátrai, Mariann Berhés, Luca Panka Molnár, Ádám Deák, Zoltán Tóth, István László, Norbert Németh, Béla Fülesdi

**Affiliations:** 1 Department of Anesthesiology and Intensive Care, Faculty of Medicine, University of Debrecen, Móricz Zsigmond Str. 22, 4032 Debrecen, Hungary; ujhelyi.bala@gmail.com (B.U.); bermarjan@yahoo.co.uk (M.B.); lacipityu@gmail.com (I.L.); 2 Department of Operative Techniques and Surgical Research, Faculty of Medicine, University of Debrecen, Móricz Zsigmond Str. 22, 4032 Debrecen, Hungary; matrai.adam@med.unideb.hu (Á.A.M.); luca.panka@hotmail.com (L.P.M.); deak.adam@med.unideb.hu (Á.D.); nemeth@med.unideb.hu (N.N.); 3 Department of Medical Microbiology, Faculty of Medicine, University of Debrecen, Nagyerdei Str. 98, 4032 Debrecen, Hungary; toth.zoltan@med.unideb.hu

**Keywords:** IgM-enriched immunoglobulin, PiCCo monitoring, *Escherichia coli*, experimental sepsis, hemodynamics

## Abstract

**Background:** Current sepsis guidelines recommend the best supportive treatment for severe sepsis, but they are limited on the effectiveness of immunomodulatory therapies. Recent data suggest that IgM-enriched immunoglobulin preparations may decrease mortality, but the exact pathomechanism remains unknown. The present experimental study aims to test the hypothesis that IgM-enriched immunoglobulin may improve hemodynamics in E-coli-induced severe sepsis. **Subjects and methods:** Sepsis was induced in the *E. coli* bacteriemia (*n* = 8), *E. coli*-parallel Pentaglobin treatment (PR-PG; *n* = 8), and *E. coli*-delayed Pentaglobin treatment (D-PG; *n* = 8). Sepsis was induced in the sepsis, PR-PG, and D-PG groups by infusing 38 mL of an *E. coli* suspension (2.5 × 10^5^/mL) over 3 h. The PR-PG group received a 0.75 g/kg Pentaglobin bolus over 20 min concurrently with the start of *E. coli* infusion. The D-PG group was given a 0.67 g/kg Pentaglobin bolus one hour after starting *E. coli*, followed by a continuous infusion at 0.02 g/kg/h for 240 min. Hemodynamic parameters were monitored every 2 h using a pulse contour cardiac output monitoring technique (PiCCo™). **Results:** Heart rate increased in all groups to varying extents. Mean arterial pressure (MAP) remained stable in controls but declined in untreated sepsis. Both Pentaglobin-treated groups showed higher MAP than untreated septic animals. Mild cardiac index increases occurred in controls and untreated sepsis, whereas the treated groups maintained a consistently elevated CI after Pentaglobin administration. Systemic vascular resistance index (SVRI) transiently increased in controls before normalizing, while untreated septic animals experienced continuous SVRI decline. Treated animals showed an initial transient SVRI rise followed by a decline; yet, SVRI remained higher than in untreated sepsis. **Conclusions:** IgM-enriched immunoglobulin led to a slight stabilization of some hemodynamic parameters, probably due to the reduced extpnfiravasation of fluids into the interstitium and, hence, had an effect on preload.

## 1. Introduction

Despite intensive clinical research in recent decades into treatments that can significantly reduce the mortality of severe sepsis, none of the therapeutic methods have actually lived up to expectations [1,2]. Although early initiation of appropriate antibiotic treatment improved mortality rates, it did not bring the expected significant reduction in in-hospital deaths [3,4]. The most recent Surviving Sepsis Guideline mainly contains supportive recommendations regarding the best intensive care treatment but makes cautious or negative recommendations regarding drugs (e.g., immunoglobulins) and interventions (e.g., blood purification) that may influence immune function [5].

The role of the immunoglobulin treatment in severe sepsis is a debated issue. Meta-analyses have demonstrated a reduction in mortality with intravenous immunoglobuline, IVIG (RR 0.73; 95% CI 0.51–0.91) and IgM-enriched immunoglobulin (RR 0.69; 95% CI 0.55–0.85); however, the quality of evidence is low because of the heterogeneity of the previous clinical trials [6,7]. In a recent meta-analysis, Pan et al. reported reduced intrahospital mortality and shorter hospital stays in patients with immunoglobulin treatment, and the analysis also proved that the efficacy of the IgM-enriched form may be superior to the standard IVIG therapy [8]. A systematic review also proved this observation and found the most consistent mortality reduction when administered early in hyperinflammatory phases [9,10].

It has been observed that septic patients have lower circulating B lymphocyte and immunoglobulin M (IgM) levels compared to non-septic patients [11,12,13]. Additionally, polyclonal immunoglobulin has been shown to reduce mortality in patients with low IgM levels [14,15,16], with Gram-negative or early septic shock [17,18,19], and with post-operative abdominal sepsis [20].

In experimental models, it has been documented that IgM-enriched immunoglobulin enhances complement inhibitory capacity compared to IVIG in a rat sepsis model [21] and that IgM-enriched immunoglobulin reduces inflammation and improves microcirculation compared to IVIG treatment in an endotoxin-induced hamster sepsis model [22]. Although small-animal sepsis models are suitable for studying cytokine changes in sepsis, large-animal models may be needed to follow the hemodynamic processes involved. Recent studies using intravenously administered *E. coli*-induced sepsis without any intervention (fluid therapy or vasoconstrictors) in pigs described the macro- and microcirculatory hemodynamic changes [23,24]. Data on the possible effect of IgM-enriched immunoglobulin on the hemodynamics in *E. coli*-induced sepsis are scarce.

The aim of this study was to verify whether IgM-enriched immunoglobulin affects the hemodynamic early stage of *E. coli*-induced experimental sepsis.

## 2. Materials and Methods

### 2.1. Experimental Animals and Protocol

The experiment was conducted in accordance with the European Union Directive (EU Directive 63/2010) and the Hungarian Animal Welfare Act (Act XXVIII of 1998 on the Protection and Welfare of Animals) and with the approval of the Animal Welfare Committee of the University of Debrecen (permission registration number: 18/2023/UDCAW). Ventilation (15–20 air changes per hour) and heating (central and underfloor heating) were provided in the animal housing at temperatures between 22 and 26 °C, depending on the animals’ body weight. Extreme and sudden fluctuations in humidity were avoided. The animals were given a feed mix appropriate to their species and drinking water was provided by a self-drinking system.

Experimental animals were divided into four different treatment arms:

1. Control group: The control group received only volume replacement therapy.
2. *E. coli* bacteriemia group: this group only received *E. coli* suspension.
3. *E. coli* parallel Pentaglobin (PG) group: animals received IgM-enriched immunoglobulin concomitantly with *E. coli* suspension.
4. *E. coli* delayed PG group: administration of Pentaglobin was started 60 min after the start of the *E. coli* suspension.

Thirty healthy 12-week-old juvenile female pigs were used in our experiment. Randomization was based on envelope randomization. The control group (*n* = 6) had an average weight of 24.5 ± 2.9 kg and a length of 91.3 ± 2.9 cm. The *E. coli* bacteriema group (*n* = 8) had an average weight of 20 ± 2.5 kg and a length of 88 ± 5.4 cm. The *E. coli* PG parallel group (*n* = 8) had an average weight of 22.3 ± 3.6 kg and a length of 88.4 ± 4.5 cm. The *E. coli* delayed PG group (*n* = 8) had an average weight of 20.3 ± 1.5 kg and a length of 84.8 ± 2.8 cm.

The experiments were performed under general anesthesia. Following premedication with azaperone (2 mg/kg i.m.; Stresnil, Elanco GmbH, Cuxhaven, Germany), induction was performed with intramuscular administration of 15 mg/kg ketamine (CP-Ketamine hydrochloride 10%) and 1 mg/kg xylazine (CP-Xylazine hydrochloride 2%). Maintenance of permanent anesthesia was provided by i.v. 1 mg/kg xylazine—10 mg/kg ketamine. As in some animals, ketamine may cause muscle tremors and tensor rigidity after anesthetic induction. In these cases, 2 mg/kgBW diazepam (Diazepam 5 mg/mL, AS Grindeks, Riga, Latvia) was administered to decrease the side effects and to ensure animal welfare. After induction, endotracheal intubation was performed, followed by pressure-assisted mechanical ventilation (Aeonmed Respiratory Ventilator VG70, Beijing Aeonmed Co., Ltd., Beijing, China). Mechanical ventilation aimed to provide an arterial partial oxygen tension of around 100 ± 10 mmHg and a partial carbon dioxide tension of around 40 ± 5 mmHg. After surgical preparation of the external jugular vein and the right femoral artery, a PiCCo arterial cannula (Getinge PiCCo Catheter 4F, 16 cm, Pulsion Medical System SE, Feldkirchen, Germany) was inserted into the artery, and a central venous cannula (Certofix Duo 7F, 20 cm, B. Braun Trading Ltd., Budapest, Hungary) was inserted into the vein for blood sampling, hemodynamic measurements, and treatment administration. A suprapubically inserted cystostomy bladder catheter was used to measure hourly urine output.

Volume replacement was used to mitigate the hypovolemic state seen in sepsis. In the control, *E. coli* bacteriemia, and PG-treated groups, pigs were given physiological saline (“Baxter” Sodium chloride 0.9%, pH = 4.5–7, osmolarity: 308 mOsm/L, Baxter Hungary Kft., Budapest, Hungary) intravenously at a rate of 10 mL/kg/h. The control group received only volume replacement therapy, whereas animals of the other three groups received *E. coli* suspension. In the *E. coli* bacteriemia, *E. coli*- parallel PG and *E. coli* delayed PG groups, sepsis induction was identical in all experimental animals.

The *Escherichia coli* (*E. coli*) culture (2.5 × 10^5^/mL; ATCC 25922, Department of Medical Microbiology, Faculty of Medicine, University of Debrecen) was administered by continuous intravenous infusion dissolved in physiological saline as follows: 2 mL for the first 30 min, followed by 4 mL for the next 30 min, and the remaining 32 mL of bacterial culture was given to the pigs for 120 min. In total, the animals received 9.5 × 10^6^ total *E. coli* in 180 min.

**Figure 1:**
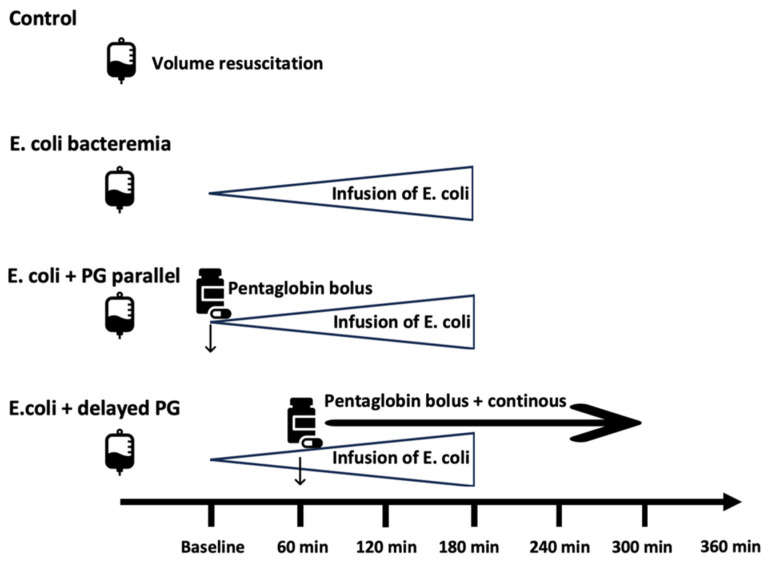
Experimental design of the study. *E. coli* indicates *Escherichia coli*; PG indicates Pentaglobin; min indicates minutes.

In the *E. coli* parallel PG group, animals received a 20 min bolus infusion of 0.75 g/kg IgM-enriched immunoglobulin concomitantly with *E. coli*. In the *E. coli* delayed PG group, a 0.67 g/kg Pentaglobin bolus was given one hour after starting *E. coli*, followed by a continuous infusion at 0.02 g/kg/h for 240 min. The volume of immunoglobulin infusion was subtracted from the volume of maintenance physiological saline so that the total volume obtained corresponded to 10 mL/kg/h. Figure 1 depicts the design of the study.

Animals from all four groups were monitored for 6 h; none of the animals were lost during the follow-up period. At the end of the experiment, the pigs were euthanized by an overdose i.v. administration of pentobarbital sodium (90 mg/kg, Release, WDT-Wirtschaftsgenossenschaft deutscher Tierärzte eG, Garbsen, Germany). No other drug (including antibiotic treatment) or therapeutic interventions were used during the study except intravenous anesthesia, physiological saline, *E. coli* suspension, immunoglobulin, and pressure-assisted ventilation.

### 2.2. Laboratory Tests

At each phase of the experiments, blood was taken for determining laboratory parameters referring to the affection of the different organs. An EPOC Blood Analysis System (Siemens Healthineers AG, Erlangen, Germany) was used for determining partial pressure of oxygen (in mmHg) in the arterial blood. Additionally, lactate (expressed in mmol/L) and serum creatinine (expressed in µmol/L) were measured using the same analyzer. Platelet count was measured using a Sysmex K-4500 (TOA Medical Electronics Co., Ltd., Kobe, Japan) hematology analyzer. PaO_2_/FiO_2_ ratio was calculated offline based on the fraction of inspired oxygen and the measured partial pressure of oxygen.

### 2.3. Hemodynamic Measurements

Hemodynamic changes were measured using a device based on transpulmonary thermodilution and pulse contour analysis (PiCCo™; PULSION Medical Systems AG, Munich, Germany). Measurements were performed using cannulas inserted into the right external jugular vein and right femoral artery. During the thermodilution measurements, 3 × 10 mL (T = 8 °C) of physiological saline was injected into the right venous catheter every two hours. A thermodilution curve was plotted from the temperature variation on the monitor. Using the area under the curve, pulse, and calculated aortic compliance, we monitored the cardiac output from heartbeat to heartbeat. Among the hemodynamic parameters were cardiac index [CI (mL/min/m^2^)], global end-diastolic volume index [GEDVI (mL/m^2^)], intrathoracic volume index [ITBVI (mL/m^2^)], extravascular lung water index [EVLWI (mL/kg)], systemic vascular resistance index [SVRI (dyn × s × cm^5^ × m^2^)], stroke volume variability [SVV (%)], and pulse pressure variability [PPV (%)]. Heart rate [HR (1/min)] and mean arterial pressure [MAP (mmHg)] were monitored invasively via a catheter placed in the femoral artery. All conditions for interpretation of PPV/SVV were met during the experiments, including sinus rhythm, low tidal volumes during mechanical ventilation and low PEEP.

### 2.4. Statistical Analysis

Statistical analyses were performed using GraphPad Prism version 9.1.2 (GraphPad Software, San Diego, CA, USA). Data are presented as mean ± S.D. (standard deviation). The normality of the data distribution was tested using the Kolmogorov–Smirnov test and, accordingly, differences within groups were compared using repeated measures ANOVA followed by Dunnett’s multiple comparisons test or Friedman test. Differences between groups were compared using independent-sample *t*-test/Mann–Whitney U test or one-way ANOVA followed by Tukey multiple comparisons test/Kruskal–Wallis test. The significance level was defined at *p* < 0.05.

## 3. Results

The main physiological parameters in the four experimental groups are summarized in Table 1. PaO_2_/FiO_2_ showed a decreasing tendency during the course of the experiments in all experimental groups. The most marked decrease was found in the *E. coli* delayed PG group. Mean arterial pressure (MAP) gradually decreased only in the *E. coli* bacteriemia group and in the group of parallel administration of *E. coli* suspension and Pentaglobin at six hours after initiation of the bacterial infusion. In the other groups (control and *E. coli* delayed PG group), the MAP remained relatively stable during the experiments. A discrete decrease in platelet counts was observed in all experimental groups that received *E. coli* suspension but not in controls. A stepwise increase in the serum creatinine levels was observed in all experimental groups but not in control animals. A mild elevation in serum lactate levels was observed in all groups during the course of the experiments.

### 3.1. Model Validation in the Control and Untreated E. coli Bacteriemia Groups

**Figure 2:**
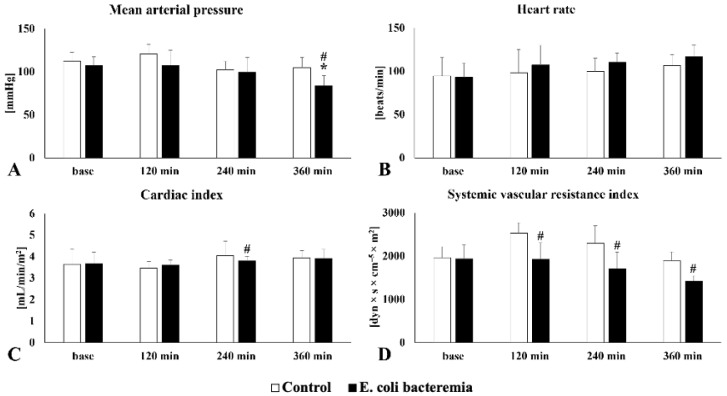
Changes in mean (**A**) arterial pressure, (**B**) heart rate, (**C**) cardiac index, and (**D**) systemic vascular resistance index in control and *E. coli* bacteriemia groups. Mean ± S.D.; *p* < 0.05; * indicates comparison with baseline; # indicates comparison to control group. Global end-diastolic volume index (GEDVI), intrathoracic blood volume index (ITBVI), extravascular lung water index (EVLWI), stroke volume variation (SVV), pulse pressure variation (PPV).

In the control group, a nearly constant mean arterial pressure was observed, whereas, in the *E. coli* bacteriemia group, a decrease was observed, which was significant at 360 min (*p* = 0.0177 compared to baseline; *p* = 0.0153 compared to control). A progressive increase in heart rate was observed in the control and *E. coli* bacteriemia groups, but higher values were obtained in the animals that received *E. coli*. The cardiac index, after an initial decline at 120 min, slightly increased during the course of the experiments in the control and bacteriemia groups. This was significant in the *E. coli* bacteriemia group compared to the control group at 240 min (*p* = 0.0286). Systemic vascular resistance returned to baseline after a transient increase in the control group. A steady decrease was observed in the bacteriemia group that was significantly lower from 120 min onward compared to the control group (120 min: *p* = 0.033, 240 min: *p* = 0.046, 360 min: *p* = 0.006) (Figure 2).

No significant differences were observed between the control and *E. coli* bacteriemia groups in GEDVI and ITBVI. For EVLWI, higher values were measured from minute 240 onward in the *E. coli* bacteriemia group compared to the control group. For SVV and PPV, higher values were observed in untreated septic animals compared to controls. For SVV, significantly higher values were measured at 240 min in the untreated sepsis group compared to the control group (*p* = 0.024) (Table 2).

### 3.2. Comparison of Hemodynamic Changes in E. coli Bacteriemia Group and PG-Treated Animals

**Figure 3:**
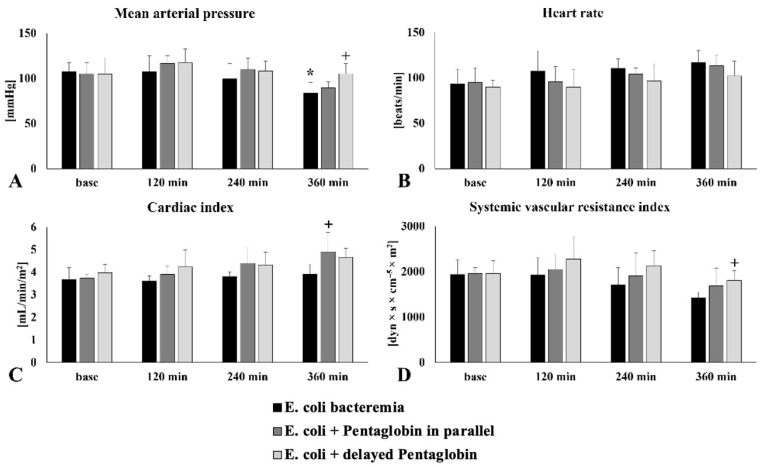
Changes in mean (**A**) arterial pressure, (**B**) heart rate, (**C**) cardiac index, and (**D**) systemic vascular resistance index in untreated sepsis and Pentaglobin-treated (*E. coli* PG in parallel and *E. coli* + delayed PG) groups. Mean ± S.D.; *p* < 0.05; * vs. baseline, + vs. untreated sepsis.

In the PG-treated groups, consistently higher MAP values were obtained compared to the untreated septic group, which was significant at 360 min in the *E. coli* delayed PG group (*p* = 0.0058 vs. untreated sepsis). In the *E. coli* parallel PG group, a plateau was observed at 60–180 min, followed by a decrease in MAP. Similarly, in the *E. coli* delayed PG group, a plateau was observed at 120–240 min. Increases in heart rate were also observed in PG-treated animals, but values were lower compared to *E. coli* bacteriemia. Lower values were also observed in the delayed PG treatment group compared to the parallel PG group. In PG-treated groups, higher CI values were obtained after starting immunoglobulin compared to the *E. coli* bacteriemia. group. The CI increase started at 60 min (not shown in the figure) in the *E. coli* parallel PG group and at 120 min in the *E. coli* delayed PG group. Significant differences were observed at 360 min in the *E. coli* parallel PG group compared to untreated septic animals (*p* = 0.0243). A decrease was observed after a temporary increase in the treated animals (*E. coli* parallel PG 60 min, *E. coli* delayed PG 120 min), but these values were higher throughout compared to untreated septic animals. The post-PG group showed significantly higher values at 360 min (*p* = 0.0483 vs. untreated sepsis) (Figure 3).

Higher values were obtained for GEDVI and ITBVI in both treated groups compared to untreated septic animals. EVLWI showed an increase in both treated and untreated groups, but the highest values were measured in the untreated sepsis group. For treated animals (*E. coli* parallel PG and *E. coli* delayed PG), lower values of SVV and PPV were observed throughout compared to untreated septic animals. SVV in the *E. coli* parallel PG group was significantly lower at both 120 (*p* = 0.0071) and 240 min (*p* = 0.0061) compared to the *E. coli* septicemia group. In the *E. coli* delayed PG group, a significant difference in SVV was observed only at 240 min (*p* = 0.025 vs. *E. coli* septicemia). The PPV was significantly higher in the untreated sepsis group compared to both treated groups from minute 120 until the end of the study (*E. coli* parallel PG at 120 min: *p* = 0.009; 240 min: *p* = 0.02; 360 min: *p* = 0.02; *E. coli* delayed PG at 120 min: *p* = 0.006; 240 min: *p* = 0.03; 360 min: *p* = 0.019) (Table 3).

## 4. Discussion

In the present study, we assessed the hemodynamic consequences of *E. coli*-induced sepsis in pigs. The development of sepsis was followed based on the main parameters of the SOFA score (Sequential Organ Failure Assessment), which is recommended for the description of the severity of sepsis [5]. As some of the parameters (such as GCS) could not be assessed during the present study, we chose to monitor the following parameters throughout the course of the experiments: PaO_2_/FiO_2_ ratio for the description of respiratory changes, mean arterial pressure (MAP values), platelet count, serum creatinine concentration and serum lactate concentration. The changes in these parameters during the course of the experiments supported the diagnosis of the developing sepsis. A significant decrease in mean arterial pressure and systemic vascular resistance, along with an increased stroke volume variation, was observed in the animals that received *E. coli* suspension as compared to non-septic pigs. These observations were in line with previous similar reports [24,25]. We found that IgM-enriched immunoglobulin treatment in this experimental setting contributed to higher mean arterial pressure and higher cardiac index values compared to non-treated animals. A tendency toward a higher systemic vascular resistance index was also consequently observed in the Pentaglobin groups (administered either in parallel to sepsis induction or after sepsis induction). It has to be noted, however, that a statistically significant difference has been observed only between untreated septic animals and the post-sepsis induction PG group at 360 min of follow-up. Pulse pressure variation (PPV) and stroke volume variation (SVV) parameters were both significantly lower in Pentaglobin-treated animals. It is known from previous observations that a decrease in PPV may be an indicator of the optimalization of the fluid status [26]. Because all experimental groups received the same amount of fluid infusions, we hypothesize that IgM-enriched immunoglobulin may have decreased the leakage of the fluid in the interstitial space and/or may have caused an increase in systemic vascular resistance and, hence, an increase in the circulating blood volume.

It is widely accepted that endothelial damage plays a crucial role in the development of sepsis syndrome [27]. The systemic release of cytokines, such as TNF-alpha and IL-beta, has been documented to induce an upregulation of the endothelial surface antigens, with subsequent leukocyte adherence and microcirculatory dysfunction [28]. Previous experimental studies have already proven the attenuating effect of IgM on microvascular perfusion failure [22].

The pathophysiology of the beneficial effect of IgM-enriched polyvalent immunoglobulin is not completely understood. It has been shown that immunoglobulins contain high-affinity neutralizing antibodies against IL-1, IL-6, and TNF-alpha, and they downregulate the synthesis of these cytokines, leading to the stabilization of the endothelial junctions [29,30]. Additionally, it has been proposed that immunoglobulins have a neutralizing effect on microbial toxins and pro-inflammatory cytokines, exert and an anti-inflammatory action, inhibit complement activation and may reduce the microcirculatory dysfunction in sepsis [22,31]. Based on experimental observations, intravenous immunoglobulins are capable of significantly reducing leukocyte adhesion and normalizing capillary diffusion [22].

The original concept of including a group with the parallel administration of *E. coli* suspension and Pentaglobin was to assess the early anti-inflammatory effects of Pentaglobin (such as increasing the clearance of endotoxins and decreasing bacterial adherence, invasion and migration). The pathophysiologic rationale for this was supported by the previous results of Barrett-Due et al. [25]. They reported on a binding of the polyvalent immunoglobulins to the LPS in the early course of bacterial sepsis using a similar experimental model.

It has to be noted that, in a previous porcine model of experimental sepsis, besides the immunomodulatory effect, a delayed hemodynamic effect of IgM-enriched immunoglobulin was observed in treated compared to non-treated septic animals [31]. Similar to our observations, they also documented a significant decrease in mean arterial pressure, an increased pulse rate and a decreased systemic vascular resistance. Although other detailed hemodynamic parameters are not available from that study, the percent change in the plasma volume was consistently higher in the Pentaglobin group until the end of the experiments (240 min), which corresponds to the decreased pulse pressure variation in the present study and may refer to an improved microcirculation.

Several limitations of this study have to be mentioned. The most important limitation of the present study is its translational nature; the results of animal experiments cannot be directly transferred to clinical practice. We induced sepsis by administering intravenous *E. coli* that may represent a certain Gram-negative form of fulminant sepsis but may not be typical for other Gram-negative or Gram-positive forms of severe sepsis. Although pre-treatment with immunoglobulins may point to a mechanism of action, it is unrealistic in clinical practice; this must be considered a “bench-to-bedside” limitation. Delayed pentaglobin treatment was initiated at a certain time point of the experiments, which is also not the case in clinical practice. However, as we intended to standardize our experiments, we finally decided to administer immunoglobulin at certain time points that were not based on clinical signs. We believe that, despite these limitations, the present study added some new information to better understanding the pathophysiological processes and hemodynamic changes in *E. coli*-induced sepsis.

## 5. Conclusions

In the present study, we found that IgM-enriched immunoglobulin administered either before or after the induction of sepsis may improve hemodynamic status by increasing peripheral vascular resistance and decreasing pulse pressure variation in *E. coli*-induced sepsis. These observations may refer to a protective effect of the immunoglobulin at the microvascular level. The results are mainly of pathophysiological value, and further studies should prove whether this effect contributes to better outcomes in human sepsis.

## Figures and Tables

**Table 1:** Main physiological parameters during the course of the experiments in the four groups. Means and standard deviations are presented; %—changes from the baseline are shown in parenthesis. PaO_2_ indicates arterial partial pressure of oxygen; FiO_2_ indicates fraction of inspired oxygen; MAP indicates mean arterial pressure.

		Control	*E. coli* Bacteriemia	*E. coli* + Pentaglobin in Parallel	*E. coli* + Delayed Pentaglobin
PaO_2_/FiO_2_ (mmHg)	baseline	780.3 ± 137.8	681.6 ± 256.5	706.9 ± 230.4	712.9 ± 174.3
	2 h	618.1 ± 46.1 (−26%)	630.9 ± 156 (−8%)	757.3 ± 493.4 (+7%)	591.2 ± 150.7 (−20%)
	4 h	652.1 ± 120.4 (−19%)	538.1 ± 83.8 (−26%)	502.8 ± 114.7 (−40%)	523.6 ± 112.3 (−36%)
	6 h	588.9 ± 89.2 (−32%)	471.4 ± 95.7 (−44%)	541.8 ± 201.4 (−30%)	444.3 ± 75 (−60%)
MAP (mmHg)	baseline	112.2 ± 10.3	107.1 ± 10.3	105 ± 12.6	104.8 ± 17.9
	2 h	120.6 ± 10.9 (+7%)	107.4 ± 17.7 (+1%)	116.5 ± 8.7 (+10%)	117.1 ± 15.5 (+11%)
	4 h	102 ± 9.8 (−10%)	99.4 ± 17.4 (−7%)	109.5 ± 12.9 (+5%)	108 ± 11.0 (+3%)
	6 h	104.5 ± 11.9 (−7%)	83.8 ± 11.5 (−27%)	89.7 ± 6.7 (−17%)	105 ± 11.5 (+0.2%)
Platelets (G/L)	baseline	502.6 ± 110.6	540.2 ± 130.9	494.2 ± 158.5	462.5 ± 115.3
	2 h	462.5 ± 175.4 (−9%)	494.8 ± 105.4 (−9%)	439.9 ± 128.6 (−12%)	383.8 ± 143.7 (−20%)
	4 h	484.8 ± 134.6 (−4%)	474.9 ± 64.6 (−13%)	432.6 ± 124.6 (−14%)	402.6 ± 131.1 (−14%)
	6 h	484.3 ± 134.8 (−4%)	485.9 ± 81.6 (−11%)	411.8 ± 111.9 (−20%)	375.8 ± 128.4 (−23%)
Creatinine (µmol/L)	baseline	86.8 ± 29.3	73.4 ± 35.1	85.9 ± 13.5	84.6 ± 14.9
	2 h	88.6 ± 23.9 (+2%)	92.1 ± 21.2 (+20%)	89.6 ± 9.1 (+4%)	83.4 ± 13.5 (−1%)
	4 h	93.7 ± 22.6 (+7%)	84.3 ± 15.2 (+13%)	93.1 ± 15.7 (+8%)	97.8 ± 12.3 (+13%)
	6 h	93.3 ± 15.9 (+7%)	110.7 ± 31.9 (+34%)	108.2 ± 25.9 (+21%)	117.2 ± 19.5 (+28%)
Urine output (ml/h)	baseline	162.5 ± 185.1	132.5 ± 133	126.1 ± 81.2	80 ± 40.4
	2 h	100 ± 32.2 (−62%)	99.3 ± 51.9 (−33%)	140 ± 120.8 (+10%)	132.3 ± 62.7 (+40%)
	4 h	220 ± 107.7 (+26%)	221.9 ± 95.8 (+40%)	170 ± 125.6 (+26%)	153.3 ± 66.2 (+48%)
	6 h	185 ± 76.4 (+12%)	198.1 ± 81.4 (+33%)	239.6 ± 160.2 (+47%)	207.2 ± 94.8 (+61%)
Lactate (mmol/L)	baseline	0.84 ± 0.18	1.05 ± 0.49	0.84 ± 0.24	0.91 ± 0.24
	2 h	1.01 ± 0.11 (+17%)	1.19 ± 0.51 (+12%)	0.93 ± 0.41 (+10%)	1.27 ± 0.28 (+28%)
	4 h	0.96 ± 0.19 (+12%)	1.31 ± 0.49 (+20%)	1.11 ± 0.32 (+24%)	1.09 ± 0.38 (+17%)
	6 h	1.14 ± 0.21 (+26%)	1.41 ± 0.26 (+26%)	1.12 ± 0.48 (+25%)	1.06 ± 0.39 (+14%)

**Table 2:** Changes in global end-diastolic volume index (GEDVI), intrathoracic blood volume index (ITBVI), extravascular lung water index (EVLWI), stroke volume variation (SVV) and pulse pressure variation (PPV) parameters in control and *E. coli* bacteriemia groups.

Variables		Baseline	120 min	240 min	360 min
GEDVI [mL/m^2^]	Control	661.2 ± 71.4	634.7 ± 42.6	603.7 ± 94.1	595 ± 52.2
	*E. coli* bacteriemia	653 ± 58.4	628.9 ± 143.4	604.3 ± 120.3	612.4 ± 95.8
ITBVI [mL/m^2^]	Control	826.8 ± 89.5	793.7 ± 52.9	754.8 ± 117.4	743.8 ± 64.8
	*E. coli* bacteriemia	838.2 ± 73.4	786.6 ± 179	755. 8 ± 150.3	766.1 ± 119.9
EVLWI [mL/kg]	Control	18.7 ± 1.5	18.4 ± 2.8	18.7 ± 3.0	18.5 ± 2.7
	*E. coli* bacteriemia	18.2 ± 2.2	17.7 ± 1.5	21.4 ± 4.8	21.6 ± 4.2
SVV [%]	Control	14.2 ± 1.5	16.2 ± 0.9	13.5 ± 4.6	16 ± 2.9
	*E. coli* bacteriemia	14.1 ± 3.7	19 ± 3.1	19.5 ± 2.3 #	19.6 ± 4. 2
PPV [%]	Control	15.5 ± 2.08	17.25 ± 2.36	17.5 ± 3	17.75 ± 2.87
	*E. coli* bacteriemia	14.8 ± 1.64	19 ± 2.53	18 ± 1.55	19.8 ± 2.17

Mean ± S.D.; *p* < 0.05; # vs. control.

**Table 3:** Changes in global end-diastolic volume index (GEDVI), intrathoracic blood volume index (ITBVI), extravascular lung water index (EVLWI), stroke volume variation (SVV), and pulse pressure variation (PPV) parameters in untreated sepsis and Pentaglobin-treated (*E. coli* PG in parallel and *E. coli* + delayed PG) groups.

Variables		Baseline	120 min	240 min	360 min
**GEDVI [mL/m^2^]**	*E. coli* bacteriemia	653 ± 58.4	628.9 ± 143.4	604.3 ± 120.29	612.4 ± 95.8
	*E. coli* PG in parallel	667.8 ± 103.5	677 ± 116.7	657.2 ± 125.1	647.6 ± 99.5
	*E. coli* + delayed PG	696.6 ± 60.8	705 ± 101.0	711.3 ± 89.5	710.8 ± 102.5
**ITBVI [mL/m^2^]**	*E. coli* bacteriemia	838.2 ± 73.4	786.6 ± 179	755.8 ± 150.3	766.1 ± 119.9
	*E. coli* PG in parallel	835.2 ± 129. 6	846.7 ± 145.6	805.9 ± 166.6	809.4 ± 124.1
	*E. coli* + delayed PG	846.5 ± 60.7	881.3 ± 126.2	889.5 ± 112.1	888.5 ± 127.7
**EVLWI [mL/kg]**	*E. coli* bacteriemia	18.2 ± 2.2	17.67 ± 1.5	21.4 ± 4.8	21.6 ± 4.2
	*E. coli* PG in parallel	18 ± 1	17.83 ± 1.8	18. 7 ± 1.2	20 ± 2
	*E. coli* + delayed PG	17.7 ± 1.4	18.7 ± 1.5	19.2 ± 1.5	19 ± 1.5
**SVV [%]**	*E. coli* bacteriemia	14.1 ± 3.7	19 ± 3.1	19.5 ± 2.3	19.6 ± 4.2
	*E. coli* PG in parallel	14.3 ± 2.3	13.1 ± 2.2 #	13 ± 3.7 #	16 ± 5.3
	*E. coli* + delayed PG	14 ± 3.8	15.7 ± 4.3	13.6 ± 3.7 #	15.17 ± 2.3
**PPV [%]**	*E. coli* bacteriemia	14.8 ± 1.6	19 ± 2.5	18 ± 1.5	19.8 ± 2.2
	*E. coli* PG in parallel	14 ± 1.9	13.8 ± 2.8 #	13.4 ± 2.4 #	15 ± 3.6 #
	*E. coli* + delayed PG	14 ± 2.4	13.5 ± 2.3 #	13.4 ± 3.8 #	15.33 ± 1.2 #

Mean ± S.D.; *p* < 0.05; # vs. *E. coli* bacteriemia.

## Data Availability

The data presented in this study are available on request from the corresponding author.
